# Retraction of ‘DICER-dependent biogenesis of let-7 miRNAs affects human cell response to DNA damage via targeting p21/p27’

**DOI:** 10.1093/nar/gkac1148

**Published:** 2022-11-30

*Nucleic Acids Research*, Volume 43, Issue 3, 18 February 2015, Pages 1626–1636, https://doi.org/10.1093/nar/gku1368

In the first quarter of 2020 and third quarter of 2021, a reader raised concerns about DICER – and Actin Western-blot bands in Figure 4C. While an institutional investigation in 2020–2021 found no evidence of inappropriate image manipulation in this article, a consultant subsequently hired by the institution echoed the reader's concerns. The Editors subsequently scrutinised other figures and found clear evidence of inappropriate image manipulation in the second panel of Figure 2D as shown below.

In this experiment, HeLa cells were treated with siDICER with or without siRNA against p21 or/and p27 (si-p21, si-p27 or si-p21/si-p27) for 60 h, and the cells were collected for immunoblot detection. Figure 2D shows that knockdown of p21 and p27 reduces the levels of Cyclin A.


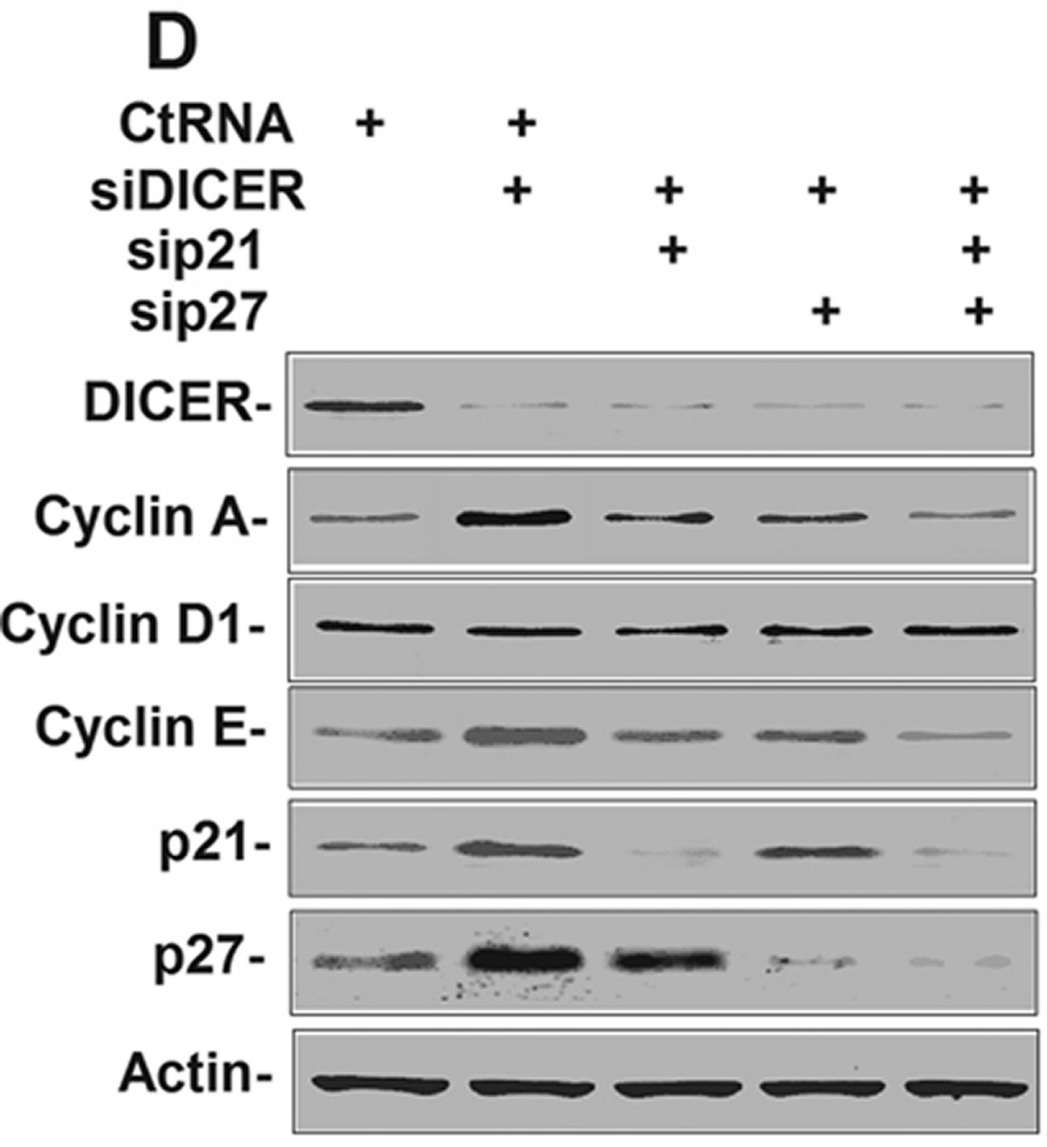


**Published Figure 2D**


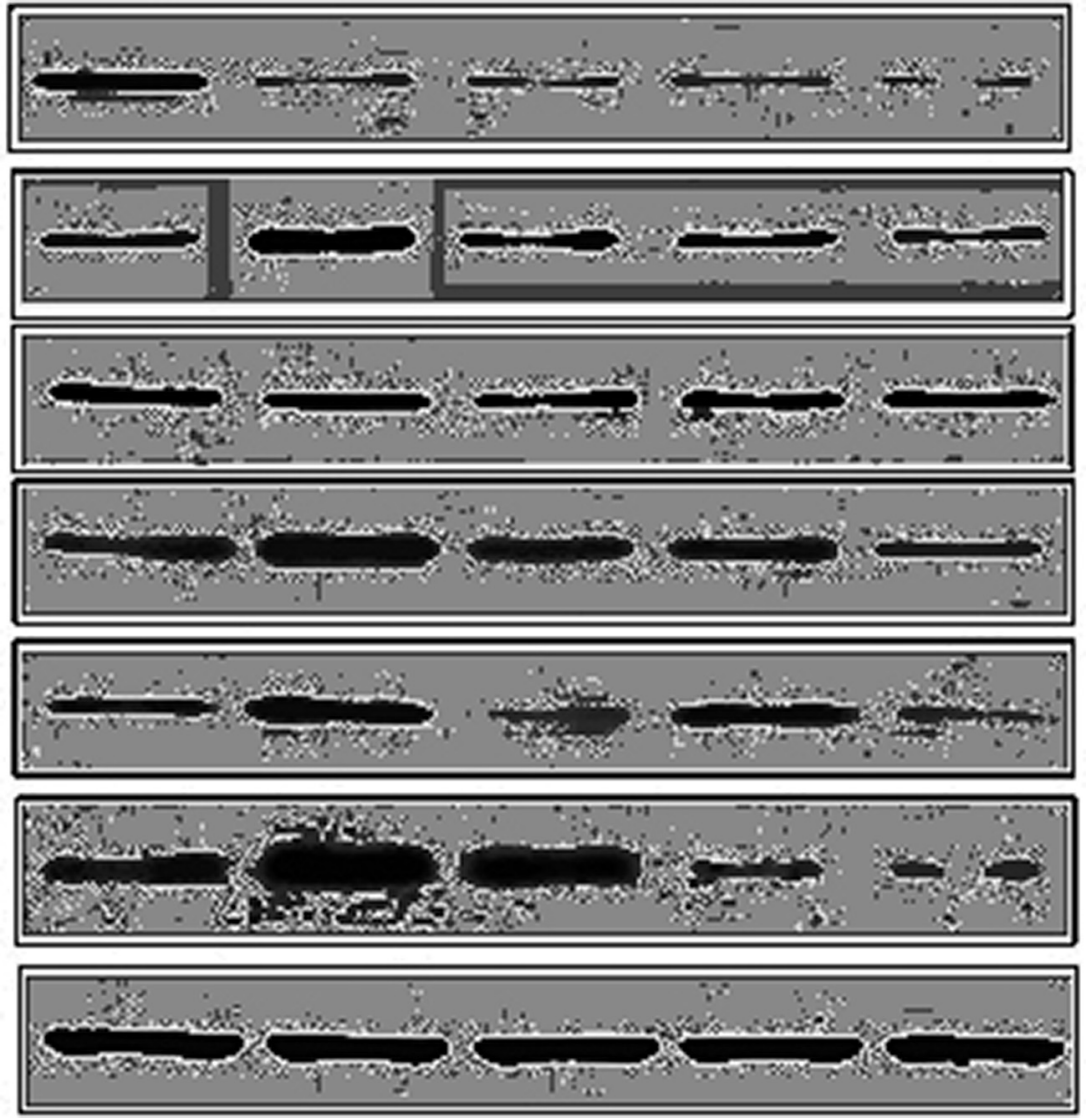


**Figure 2D after Equalize adjustment**. Signs of inappropriate image manipulation in panel 2.

The authors are unable to provide the original raw data used to produce this figure but have provided a contemporaneous experiment, shown below. The amount of loaded protein is this experiment is higher than the amount used for the NAR figure.


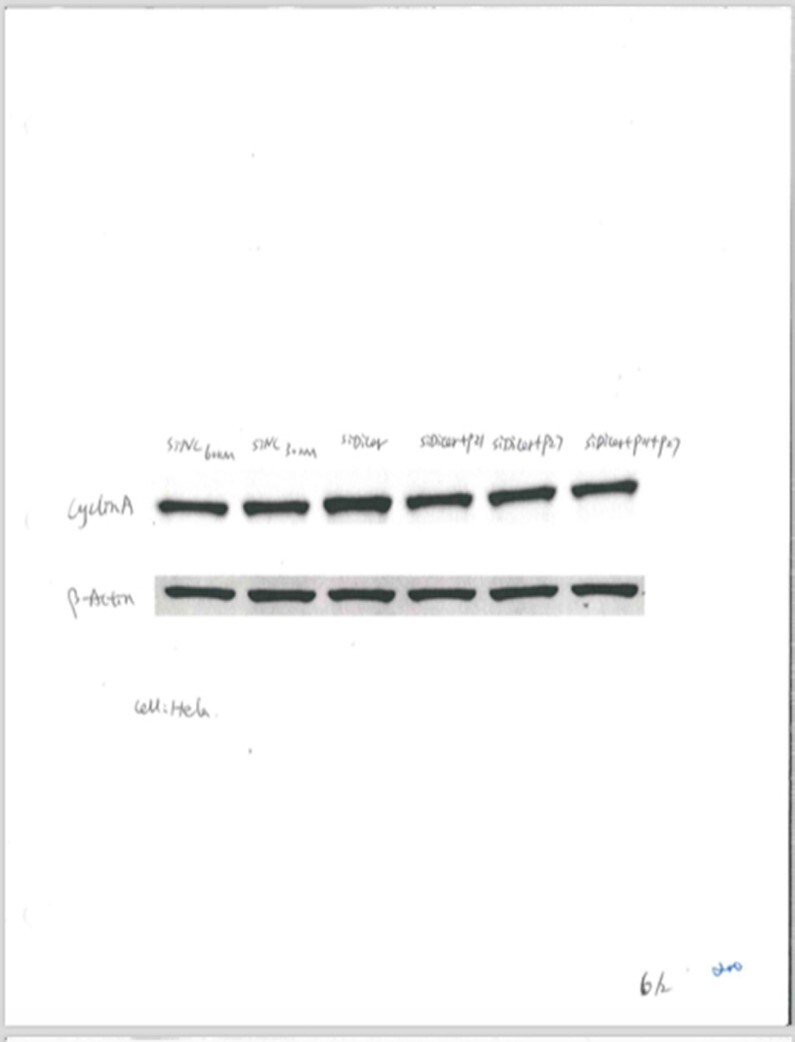


**Contemporaneous experiment with higher protein load**.

Top panel: Cyclin A. From left to right: siRNA Negative Control 60 nM, siRNA Negative Control 30 nM, siDICER alone, siDICER + sip21, siDICER + sip27, siDICER + sip21 + sip27

In conclusion, the data and corresponding Figure 2 have been inappropriately manipulated. The authors are unable to provide the original data for inspection. There may be additional issues beyond the ones reported here. Collectively, these issues render the findings unreliable, and the conclusions may no longer be valid. The Editors are retracting this article over the objections of the corresponding author.

